# Prevalence and distribution of *Aggregatibacter actinomycetemcomitans* and its *cdtB* gene in subgingival plaque of Chinese periodontitis patients

**DOI:** 10.1186/1472-6831-14-37

**Published:** 2014-04-13

**Authors:** Xiaoqian Wang, Lu Li, Mifang Yang, Ying Geng, Huiping Chen, Yan Xu, Ying Sun

**Affiliations:** 1 Laboratory of Oral Infection and Immunology, Institute of Stomatology, Nanjing Medical University, Nanjing, Jiang Su, China; 2 Department of Periodontology, College of Stomatology, Nanjing Medical University, Nanjing, Jiang Su, China

**Keywords:** *Aggregatibacter actinomycetemcomitans*, Cytolethal distending toxin, Subgingival plaque, Real-time PCR

## Abstract

**Background:**

*Aggregatibacter actinomycetemcomitans* (*A.actinomycetemcomitans*) is an important periodontal pathogen that can participate in periodontitis and other non-oral infections. The cytolethal distending toxin (Cdt) is among the virulence factors produced by this bacterium. This study was to elucidate the distribution of *A.actinomycetemcomitans* and the prevalence of its *cdtB* gene in Chinese subjects.

**Methods:**

A total of 255 subgingival samples were obtained from 30 subjects. Samples were collected from periodontal healthy sites as well as shallow, moderate and deep pockets. The absolute quantity of *A.actinomycetemcomitans* and *cdtB* gene were determined by real-time polymerase chain reaction.

**Results:**

*A.actinomycetemcomitans* was detected in 92 of 105 (87.6%) samples of aggressive periodontitis (AgP) patients, in 73 of 79 (92.4%) samples of chronic periodontitis ( CP) patients and in 5 of 71 (7.0%) samples of periodontal healthy subjects. The *cdt*B gene was detected in 72 sites (78.3%) with AgP infected with *A.actinomycetemcomitans*, 54 sites (74.0%) with CP infected with *A.actinomycetemcomitans* and none in healthy sites infected with *A.actinomycetemcomitans*. In addition, quantity of *A.actinomycetemcomitans* and *cdt* gene in samples from deep pockets were significant larger than moderate, shallow and healthy sites (P < 0.05). In comparison to CP, AgP patients were infected with increased numbers of *cdt* genotype in deep pockets (P < 0.05).

**Conclusion:**

This study suggests that the *cdtB* gene are prevalent in *A.actinomycetemcomitans*, and the distribution of *cdt* genotype strain may be correlated with AgP and serious periodontal inflammation.

## Background

Periodontitis is a chronic inflammatory disease leading to the loss of periodontal tissues, which is highly prevalent and is the major cause of tooth loss in adults. **A**ggressive periodontitis (AgP) is characterized with rapid development and serious bone resorption, generally affects younger patients than does the chronic form.

*A.actinomycetemcomitans* is a Gram-negative, non motile rod, facultative anaerobic and commensal bacterium, which has long been strongly associated with AgP and may also contribute to chronic periodontitis (CP). Apart from oral infection, this bacterium has also been responsible for some systemic infectious diseases, such as endocarditic, meningitis, osteomyelitis, glomerulonephritis and arthritis [1].

This microorganism expresses several potential virulence factors that are involved in the colonization in oral cavity, inhibition of regeneration of periodontal tissues and interference with host defense mechanisms. In 1998, Ohguchi [1] revealed that *A.actinomycetemcomitans* could produce cytolethal distending toxin (CDT), which was secreted into the bacterial culture supernatant. It is clear that CDT is encoded by three genes designated *cdtA*, *cdtB*, and *cdtC*, which are arranged as an apparent operon. These three genes specify three polypeptides designated CdtA, CdtB and CdtC with apparent molecular masses of 28, 32 and 20 kDa respectively, which form a heterotrimeric holotoxin [2-5]. CdtA and CdtC are necessary for the secretion of the toxin, while CdtB is responsible for the biologic activity [6]. CdtB has a sequence homology with mammalian DNase I, indicating a critical role for nuclease activity in host parasite interactions [7].

Among periodontal pathogenic bacteria, *A.actinomycetemcomitans* is the unique bacterium that can produce CDT. Accumulating evidences show that CDT is associated with the persistence of infection in animal models [7], increased expression of RANKL (receptor activation of nuclear factor-κB ligand) and the consequent osteoclastogenesis [3]. There was report showing that *cdtABC* was absent [8] in *A.actinomycetemcomitans* isolated in Japan [9], but Kawamoto confirmed that *cdtABC* was frequently found in the genome of *A.actinomycetemcomitans*[10].

Till now, there have been few reports on the prevalence of *cdt* genotype strain of *A.actinomycetemcomitans* in Chinese periodontitis patients. Accumulating evidences show that CDT is associated with the persistence of infection in animal models [7], increased expression of RANKL (receptor activation of nuclear factor-κB ligand) and the consequent osteoclastogenesis [3]. The association of CDT genetic diversity within *A.actinomycetemcomitans* should be better evaluated. In this study, we detected the distribution of *A.actinomycetemcomitans* and the prevalence of its putative virulence factor CDT encoding gene *cdt*B in subgingival plaque obtained from Chinese patients suffering from CP and AgP by real-time PCR, to evaluate the association of *A.actinomycetemcomitans* CDT genetic diversity and clinical features.

## Methods

### Subjects

This study was approved by the Ethical Committee of Stomatological Hospital affiliated to Nanjing Medical University, Nanjing, China. The purposes and procedures of the study were explained and informed consents were obtained from all recruits.

Participants included in the present study were recruited at the Stomatological Hospital affiliated to Nanjing Medical University, from December 2008 to March 2009. 10 CP patients, 10 AgP patients and 10 healthy subjects were recruited in this study.

The criteria for patient inclusion were as follows: (i) ethnic Han and<40 years old; (ii) no history of periodontal therapy; (iii) no systemic antibiotics or anti-inflammatory drugs taken within 3 months; (iv) healthy systemic conditions; (v) no pregnancy , and (vi) were not current users of tobacco products or nicotine replacement medication [10]. The periodontal healthy subjects had no sites with probing depth (PD) >3 mm or clinical attachment loss (CAL) >1 mm, and no more than 10% of sites with bleeding on probing (BOP).The diagnoses of CP and AgP were made based on criteria defined at the workshop sponsored by the American Academy of Periodontology (AAP) in 1999 [11-13].

Sample sites were classified as shallow, moderate or deep according to the levels of PD and CAL. The level of PD was about 3 mm or the level of CAL was 1–2 mm in shallow group; and 4–6 mm of PD or 3–4 mm of CAL in moderate group. In deep group, the level of PD was over 6 mm or CAL ≥ 5 mm [12].

### Clinical measurements

Before sampling, a complete periodontal examination was conducted to record clinical periodontal parameters by using FP32 probe (Florida probe, USA), including PD, CAL and BOP. All measurements were performed by a calibrated examiner. To avoid any contaminations that might result from bleeding on probing, clinical probing and measurement were carried out at least 7 days in advance of bacteria sampling [14].

### Sampling of subginginval bacteria plaque

8–10 samples were taken from shallow, moderate and deep sites of each enrolled patients suffering from CP and AgP, as well as from gingival sulci of healthy controls. After careful removal of supragingival plaque deposits, the sampling site was isolated with cotton rolls and gently air-dried. Then, a 30# paper point [15,16] was inserted into the pockets/gingival sulci and left in place for 30s. The paper point from each sampling site was immediately placed into an empty 1.5 ml microfuge tube. Samples for PCR analysis were stored at -80°C.

### Positive control bacterial strains

*A.actinomycetemcomitans* ATCC 29522, ATCC 29523, ATCC 24523 were grown anaerobically (75% N_2_, 10% CO_2_, 15% H_2_) at 37°C on 5% sheep blood agar plates (Oxoid, UK) enriched with haemin (5 mg/l) and menadione (1 mg/l) for 3–5 days, and then inoculated into brain heart infusion broth until grown to the late logarithmic phase of growth. The bacteria were harvested by centrifugation, washed in PBS, and re-suspended at a concentration with optical density at 690 nm of 1, corresponding to approximately 1 × 10^8^ colony-forming units (CFU)/ml [17].

### Oligonucleotide primers and TaqMan probes

Identification of conserved regions was done by multiple sequence alignment with ClustalW software based on the published 16S rDNA and *cdtB* sequences. The fluorescent dyes at the 5′ and 3′ ends of the probe were FAM (6-carboxyfluorescein; reporter) and TAMRA (6-carboxytetramethylrhodamine; quencher), respectively. All primers and probes were checked for possible cross-hybridization with bacterial genes using the database similarity search program BLAST. Primers and probes used for quantification of *A actinomycetemcomitans* 16S rDNA and *cdt* gene were shown in Table 1.

**Table 1:** Primers and TaqMan probes sequences

**Primer**	**Sequence**	**Products size**
*Aa*-F	GCTGGTCTGAGAGGATGGC	
*Aa*-R	CGAAAGAACTTTACAACCCGA	153 bp
*Aa*-P	CCTACGGGAGGCAGCAGTGG	
*cdtB*-F	ATTCTTCTGTGCTTCAATCTCG	
*cdtB*-R	GGTGATGATGGTGATGAGGTAA	151 bp
*cdtB*-P	CACAGGTGGTTCTGATGCGGTAA	

*Aa*-F, *Aa*-R and *Aa*-P stand for primers and TaqMan probe for *A.actinomycetemcomitans* 16 s rDNA; *cdtB*-F, *cdtB*-R and *cdtB*-P stand for primers and TaqMan probe for *A.actinomycetemcomitans cdtB*.

### Standard curves construction

In order to establish the quantitative assay, plasmids containing the target sequences of *A.actinomycetemcomitans* 16S rDNA and *cdt* gene were cloned using the pMD 19 T-Vector (Takara, Japan). PCR products for *A.actinomycetemcomitans* 16S rDNA and *cdt* gene were inserted into plasmid vectors respectively, and the recombinant vectors were transformed into *E. coli*. Then, the plasmids were purified with Mini BEST Plasmid Purification Kit (Takara, Japan). The purified plasmids were quantified by spectrophotometry. Standard curves were constructed by using serial diluted purified plasmids with predetermined concentrations on the basis of the linear relationship between the Ct and the logarithm of the starting gene amount. Sensitivity of the developed real-time PCR assay was evaluated by using 10^7^–10° plasmid copies of *A. actinomycetemcomitans* and *cdtB* gene (data not shown), limit of approximately 10 cells was established in the PCR reaction mixture.

### DNA extraction and Real-time PCR

Each sample was diluted in 500 μl distilled water, dispersed in vortex for 1 min, took out the paper points before DNA extraction. Then, DNA was purified by the Mini BEST Bacteria Purification Kit (Takara, Japan), and eluted in 60 μl elution buffer.

TaqMan Universal PCR Master Mix (Applied Biosystems, USA) was used for PCR analysis. The final concentration was used in a total volume of 10 μl contained 5 μl of 2 × Master Mix, 3 μl of DNA template, 0.9 mM of each primer and 0.25 mM of probe. Real-time PCR was carried out in duplicates in ABI 7900 HT system (Applied Biosystems, USA) with the following sequence: 2 min at 50°C, 10 min at 95°C and 40 cycles of 15 s at 95°C and 1 min at 60°C.

### Statistical analysis

Descriptive analysis was performed for all variables. Quantitative variables were described by mean values, standard deviations, as well as minimum and maximum values. Qualitative variables were described by absolute and relative frequencies. Comparisons were made between groups (AgP, CP and healthy subjects) for independent variables (PD) by applying analysis of variance (ANOVA). All tests were performed using SPSS for Windows Release 12.0 (USA) and P values less than 0.05 were considered significant.

## Results

### Clinical analysis

We analyzed plaque samples collected from 30 subjects following the protocols described above. This study consisted of 255 subgingival plaque samples, which were collected from 10 AgP patients, 10 CP patients and 10 periodontal healthy subjects. The study population had no previous history of smoking or periodontal treatment.

The mean values of PD, CAL and BOP (%) of all sampling sites were shown in Table 2. These results revealed that there were no statistical differences in these parameters between CP and AgP groups, while there were significant differences between either CP group or AgP group and periodontal healthy subjects.

**Table 2:** Clinical characteristics of periodontitis patients and healthy controls

	**H**	**CP**	**AgP**
Subjects	10	10	10
Sampling sites	71	79	105
Age(years-old, mean ± SD)	27.5 ± 2.1	32.5 ± 1.8	29.7 ± 2.1
Gender(male/female)	5/5	5/5	5/5
PD(mm, mean ± SD)	2.4 ± 0.8	4.1 ± 2.0 *	4.9 ± 1.9 *
CAL(mm, mean ± SD)	0	4.8 ± 2.0 *	4.5 ± 1.9 *
BOP(%, mean ± SD)	15.8 ± 0.9	30.8 ± 5.5 *	30.6 ± 7.2 *

AgP, CP and H stand for aggressive periodontitis, chronic periodontitis and periodontal healthy, respectively.

The mean probing depth, clinical attachment loss and bleeding on probing (%) were recorded in 255 sampling sites from 30 patients. * There were significant differences for mean probing depth, clinical attachment loss and bleeding on probing (%) between AgP or CP and H (P < 0.05), but there was no significant difference between AgP and CP.

### Occurrence and distribution of *A.actinomycetemcomitans*

Using real-time PCR, we examined 255 subgingival plaque samples to evaluate the levels of *A.actinomycetemcomitans* in different status of periodontal sites. Among all of the 255 subgingival samples, *A.actinomycetemcomitans* was detected from 170 (66.7%) samples. Only 5 samples (7.0%) out of the 71 subgingival samples from the periodontally healthy individuals were *A.actinomycetemcomitans* 16 s rDNA positive (Table 3). 73 of the 79 subgingival plaque samples (92.4%) from CP patients were *A.actinomycetemcomitans* 16 s rDNA positive. And among the 105 subgingival plaque samples from AgP patients, 92 (87.6%) were *A.actinomycetemcomitans* 16 s rDNA positive. There was no statistical difference of *A.actinomycetemcomitans* positive rates between CP (92.4%) and AgP (87.6%) (P > 0.05) (Table 3).

**Table 3:** **Prevalence of *A.actinomycetemcomitans *** **and *cdt *** **in all sampling sites**

	**AgP**	**CP**	**H**
Total	105	79	71
*Aa*(+)	92	73	5
*Aa*(+)/Total(%)	87.6	92.4	7.0
*cdt*(+)	72	54	0
*cdt*(+)/*Aa*(+)(%)	78.3	74.0	0
Quantity-*Aa*	2.3 ± 1.1*	2.1 ± 0.9*	0.8 ± 0.5
Quantity-*cdt*	1.6 ± 0.8**	1.3 ± 0.8***	0.4 ± 0.3

*Aa*, AgP, CP and H stand for *A.actinomycetemcomitans*, aggressive periodontitis samples, chronic periodontitis samples and periodontal healthy samples, respectively.

*There were significant differences for quantity of *A.actinomycetemcomitans* between AgP or CP and H (P < 0.05) and no significant differences between AgP and CP (P > 0.05). **Quantity of *cdt* genotype of *A.actinomycetemcomitans* from AgP samples were significantly higher than that of CP and H (P < 0.05). ***Quantity of *cdt* genotype of *A.actinomycetemcomitans* from CP samples were significantly higher than H (P < 0.05).

Mean log-transformed numbers of *A.actinomycetemcomitans* 16 s rDNA in healthy subjects, CP patients and AgP patients were 0.8 ± 0.5, 2.1 ± 0.9 and 2.3 ± 1.1 (Table 3), respectively. Higher numbers of *A.actinomycetemcomitans* were detected in samples from periodontitis sites in contrast to those from healthy subjects (P < 0.05). However, there were no significant differences between these two periodontitis groups (P > 0.05).

### Prevalence and distribution of *A.actinomycetemcomitans cdtB* gene

Out of 255 subgingival samples, *A.actinomycetemcomitans cdtB* was detected in 126 (49.4%) samples. In the 71 subgingival samples from periodontal healthy individuals, none of the samples were *cdtB* positive (Table 3). While 54 of the 73 (74.0%) *A.actinomycetemcomitans* positive subgingival plaque samples from CP patients were *cdtB* positive. Among the 92 *A.actinomycetemcomitans* positive subgingival plaque samples from AgP patients, 72 (78.3%) samples were *A.actinomycetemcomitans cdtB* positive. There was no statistical difference for the *A.actinomycetemcomitans cdtB* positive rates between CP (74.0%) and AgP (78.4%) (P > 0.05). None of the *A.actinomycetemcomitans* 16 s rDNA negative samples were positive for *cdtB.*

Levels of *cdtB* gene were also detected in 255 samples. Mean log-transformed numbers of *cdtB* in CP and AgP were 1.3 ± 0.8 and 1.6 ± 0.8 (Table 3). Samples of AgP group showed the higher numbers of *cdtB* genotype *A.actinomycetemcomitans* contrast to CP group and periodontal healthy subjects (P < 0.05).

### Association of *A.actinomycetemcomitans* and *cdt* distribution with different periodontal status

Mean log-transformed numbers of *A.actinomycetemcomitans* and *cdtB* gene in shallow, moderate and deep pockets were 1.8 ± 0.9, 2.2 ± 0.8, 3.1 ± 1.2 and 1.3 ± 0.8, 1.5 ± 0.8, 2.1 ± 1.1 (Table 4), respectively. Higher quantity of *A.actinomycetemcomitans* and *cdtB* gene in deep periodontal pockets were detected than moderate and shallow pockets (P < 0.05).

**Table 4:** **Prevalence and distribution of *A. actinomycetemcomitans *** **and *cdt *** **in periodontitis sampling sites by probing depth**

	**Shallow**	**Moderate**	**Deep**
Total	100	49	35
Quantity-*Aa*	1.8 ± 0.9	2.2 ± 0.8*	3.1 ± 1.2**
Quantity-*cdt*	1.3 ± 0.8	1.5 ± 0.8	2.1 ± 1.1**

*Mean log-transformed numbers of *A.actinomycetemcomitans* 16 s rDNA from moderate pockets samples were significantly higher than that from shallow pockets (P < 0.05).

**Mean log-transformed numbers of *A.actinomycetemcomitans* 16 s rDNA and *cdt* genotype of *A.actinomycetemcomitans* from deep pockets samples were significantly higher than that from moderate and shallow pockets (P < 0.05).

**Figure 1:**
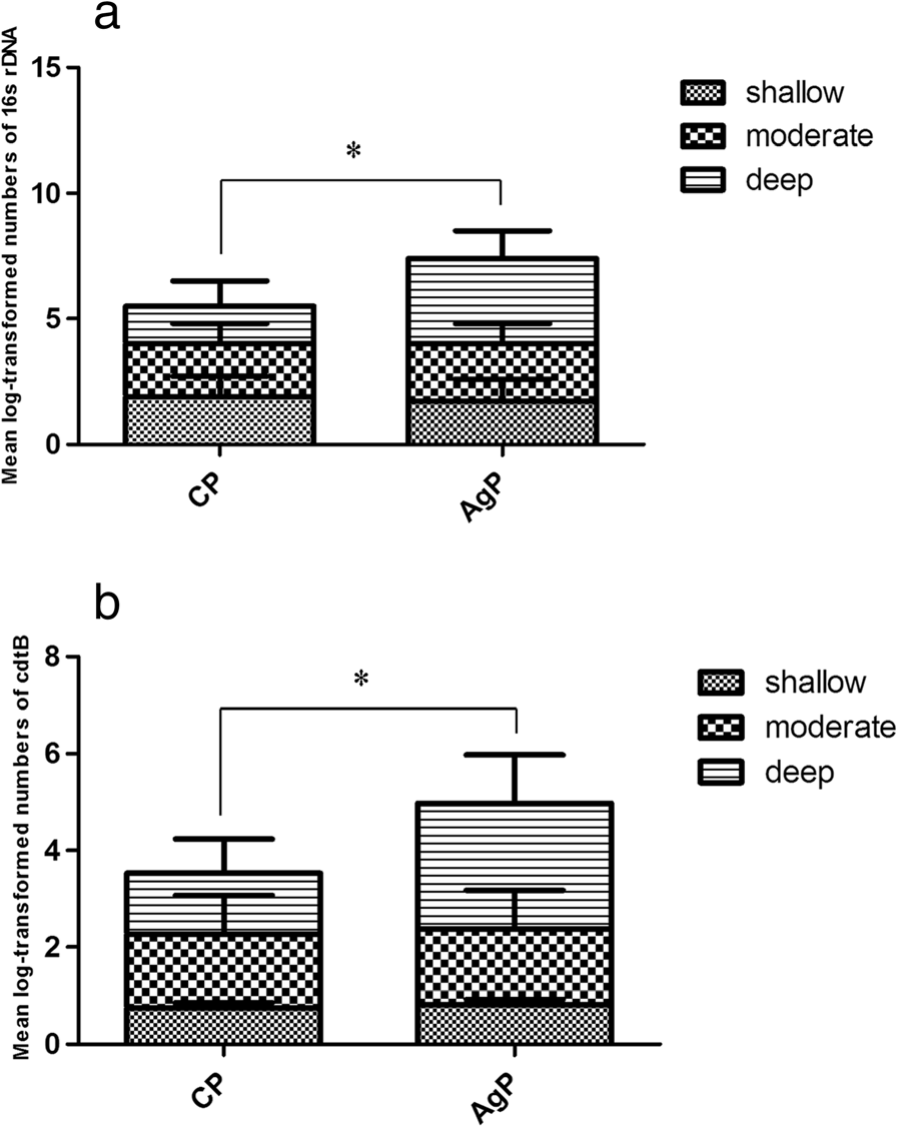
**Mean log-transformed numbers of A.actinomycetemcomitans 16s rDNA and cdtB in shallow, moderate and deep periodontal pockets. a**. Mean log-transformed numbers of *A.actinomycetemcomitans* 16 s rDNA in shallow (n = 48), moderate (n = 20), deep (n = 11) periodontal pockets from CP subjects were 1.9 ± 0.8, 2.1 ± 0.8 and 2.5 ± 1.0, respective. Mean log-transformed numbers of *A.actinomycetemcomitans* 16 s rDNA in shallow (n = 52), moderate (n = 29), deep (n = 24) periodontal pockets from AgP subjects were 1.7 ± 0.8, 2.3 ± 0.8 and 3.4 ± 1.1, respective. * The quantity of *A.actinomycetemcomitans* in deep periodontal pockets from AgP was higher than CP (P < 0.05). **b**. Mean log-transformed numbers of *A.actinomycetemcomitans cdtB* in shallow (n = 48), moderate (n = 20), deep (n = 11) periodontal pockets from CP subjects were 0.8 ± 0.1, 1.5 ± 0.8 and 1.3 ± 0.7, respective. Mean log-transformed numbers of *A.actinomycetemcomitans* 16 s rDNA in shallow (n = 52), moderate (n = 29), deep (n = 24) periodontal pockets from AgP subjects were 0.8 ± 0.1, 1.6 ± 0.8 and 2.6 ± 1.0, respective. * The quantity of *A.actinomycetemcomitans cdtB* in deep periodontal pockets from AgP was higher than CP (P < 0.05).

With respect to the various periodontal statuses, mean log-transformed numbers of *A.actinomycetemcomitans* and *cdtB* gene from CP and AgP in shallow, moderate and deep pockets were shown in Figure 1 respectively. The quantity of *A.actinomycetemcomitans* and *cdtB* gene in deep periodontal pockets from AgP was higher than CP (P < 0.05).

## Discussion

This research we employed TaqMan real-time PCR to direct target quantification *A.actinomycetemcomitans* and *cdt*B gene in different severity of periodontitis sites of AgP or CP patients.and it is effective for detecting pathogen species that are extremely difficult to culture. Therefore, we chose different periodontal sites as targets to explore the occurrence and quantity of A.actinomycetemcomitans and its CDT encoding gene in Chinese subjects and different periodontal status. *A.actinomycetemcomitans* might not be present in all oral sites in an untreated periodontitis patient [18]. Taking subgingival samples from all teeth would be the most reliable way to detect *A.actinomycetemcomitans*. However, this method is money- consuming and time-consuming which is not suitable to be used in daily practice. Sampling from the deepest pocket of each quadrant has been demonstrated to be quite reliable for detecting the subgingival presence of periodontal pathogens in untreated patients [18]. This technique was based on individual level. While one suffered from periodontitis, aggressive or chronic mode, severity of periodontal disease might differ from site to site. So we evaluated relevance of *A.actinomycetemcomitans* and its CDT encoding gene with periodontitis stuates base on “site”.

The primers chosen for detection of *A.actinomycetemcomitans* and *cdt* gene were based on *A.actinomycetemcomitans* 16 s rDNA gene and *cdtB* gene respectively. The 16 s rDNA gene has been reported to be highly conserved and this pattern for detection of *A.actinomycetemcomitans* has been well preserved [19,20]. Moreover, recent data have shown that CdtB is the main component and indispensable for the expression of CDT holotoxin activity [6,8], and the sequence of *cdtB* was also highly conserved among *A.actinomycetemcomitans* species [21]. Thus, the prevalence of CDT encoding genes was evaluated using *cdtB* specific primers and probes.

Standard curves were used in this study to evaluate the absolute quantification of *A.actinomycetemcomitans* and *cdt* gene in samples. Plasmids containing cloned target sequences were used as standard substances in quantitative PCR, which enabled the measurements more precise and steady compared to using PCR amplicon as standard substances directly [22].

Some studies have examined the prevalence of *A.actinomycetemcomitan*s in some different populations [4,13,18]. Our work is the first report on the analysis of both positive rate and absolute quantity of *A.actinomycetemcomitans* and its *cdt*B gene in Chinese periodontitis patients, and the association of the distribution of *A.actinomycetemcomitans* and *cdtB* with various periodontal status.

We found that *A.actinomycetemcomitans* and its *cdtB* gene were significantly more prevalent and with higher quantity in samples from patients suffering from AgP or CP than periodontal healthy subjects, and *A.actinomycetemcomitans* and its *cdt* gene were also more prevalent and with higher quantity in deep periodontal pockets than in moderate and shallow periodontal pockets.

Although *A.actinomycetemcomitans* is linked to the etiology of AgP, this bacterium is also found in subjects who are healthy or have other forms of periodontal disease [8]. Our results showed that only 7.0% periodontal healthy sites were *A.actinomycetemcomitans* positive; 92.4% CP samples and 87.6% AgP samples exhibited *A.actinomycetemcomitans* positive, demonstrating that the presence of *A.actinomycetemcomitans* was correlated with periodontitis.

Quantitative data showed that the amount of *A.actinomycetemcomitans* in CP and AgP samples were observably higher than in healthy samples, while no difference between CP and AgP. But the amount of *cdtB* genotype strain of *A.actinomycetemcomitans* in AgP samples were remarkablly higher than in CP and healthy samples (Table 3). These data may indicate quantitative results were more suitable to analyse the distribution of *A.actinomycetemcomitans* and its *cdtB* genotype strain*s*. The *cdtB* genotype strain of *A. actinomycetemcomitans* may be more relevant with aggressive periodontitis.

Tan and his coworkers found *A.actinomycetemcomitans* with the *cdt* genotype were at a higher frequency from sites obtained from patients diagnosed with aggressive periodontitis [23]. Our study showed the *cdtB* genotype *A.actinomycetemcomitans* were at a higher quantity from sites obtained from deep pockets. The higher occurrence and amount of this bacterium in samples with severe periodontitis status was not a surprising observation. *A.actinomycetemcomitans* CDT toxin may be similar to *H.ducreyi* CDT toxin, which may contribute to the pathogenicity of bacteria at a higher concentration [7]. In vivo studies are still needed to explore the exact pathogenic role of *A.actinomycetemcomitans* CDT in the future. Our data showed *A.actinomycetemcomitans* and its *cdt* genotype strain were prevalent in deep and moderate periodontal pockets. To move forward a single step, quantitative analysis showed *cdtB* genotype strain of *A.actinomycetemcomitans* were more prevalent in deep periodontal pockets. The quantities of *cdt* genotype strain of *A.actinomycetemcomitans* were correlated with severe forms of periodontitis (CP or AgP). Numerous studies have considered *A.actinomycetemcomitans* as an important etiological microorganism involved in AgP [24-26], however, from a new perspective, our data showed that *cdtB* genotype strain of *A.actinomycetemcomitans* was mainly found among sites with severe forms of periodontitis. *A.actinomycetemcomitans* with *cdtB* genotype may be more virulent to human periodontium.

As expected, none of the *A.actinomycetemcomitans* 16 s rDNA negative samples were positive for *cdtB*. This result confirmed that *A.actinomycetemcomitans* was the exclusive member in the oral microbial flora identified to carry and express the cytolethal distending toxin locus. Besides, there were 5 of healthy samples in our study which were positive for *A.actinomycetemcomitans* while negative for *cdt*. This may be results of minute quantity of *cdt* genotype strain of *A. actinomycetemcomitans* in this 5 subgingival plaque samples, while may be a new supporting proof for the exist of *cdt*-negative genotype strain of *A.actinomycetemcomitans* in oral cavity.

The *cdt* gene as well as *lktA* (leukotoxin A) of *A.actinomycetemcomitans* is a single copy gene [21]. However, 16 s rDNA gene may generally have 4 to 6 copies per cell (e.g., 6 copies for *E. coli*) [22]. Our results showed that within the same sample the absolute quantity of *A.actinomycetemcomitans* 16 s rDNA were 1–10 times over that of *cdtB* gene, which indicated that one periodontal site might be infected with two or even more genotypes of *A.actinomycetemcomitans* simultaneously. Some genotypes of *A.actinomycetemcomitans* possess *cdt* gene, which could express CDT activity; while other genotypes of *A.actinomycetemcomitans* are *cdt*-negative, which would be less virulent than *cdt*-positive strains. Yamano [9] reported that 89% of *A.actinomycetemcomitans* strains possessed the *cdt* gene. Another study [4] discovered that 86% of *A.actinomycetemcomitans* isolates presented complete operon of *cdt* gene and its characteristic cytotoxic activity. Tan and his coworkers showed a close association between AgP and *cdt*-positive genotype *A.actinomycetemcomitans* strains [23]. These findings suggested that not all the strains of *A.actinomycetemcomitans* possessed *cdt* gene, in other words, not every *A.actinomycetemcomitans* strains presented cytotoxic CDT activity. Similar to *A.actinomycetemcomitans* strains, *Campylobacter spp*., *C.jejuni*, *H.ducreyi* and other CDT-producing bacteria don’t express CDT activity or contain all of the *cdtABC* genes in all strains [4].

## Conclusion

Our study investigated the prevalence and distribution of *A.actinomycetemcomitans* and its *cdt* gene in subgingival plaque from Chinese periodontitis patients. The significantly increased quantities of *cdt*-positive genotype *A.actinomycetemcomitans* were found in AgP periodontal sites and in deep pockets of both CP and AgP patients, which indicated that *cdt* gene might be a potential virulence-associated gene involved in the pathogenesis of AgP and severe periodontal destruction. Our results suggested the importance of obtaining both *A.actinomycetemcomitans* titer and genotype identification in periodontitis microbiological diagnosis. Extensive studies are necessary for providing more information about the molecular pathophysiological role of *A.actinomycetemcomitans* and its CDT.

### Transparency declaration

This study is supported by grants from the National Natural Science Foundation of China (Grant No. 81170962), Project of Science and Technology Department of Jiangsu Province (Grant No. BK2011763) and Project Funded by the Priority Academic Program Development of Jiangsu Higher Education Institutions (Grant No. PAPD2011-2013).

## Competing interests

The authors declare that they have no competing interests.

## Authors’ contributions

XW and LL carried out the samples collection, periodontal examination, participated in the DNA extraction and Real-time PCR and drafted the manuscript. YG and HC participated in the sequence alignment. YX, YS participated in the design of the study. MY participated in its design and coordination and helped to draft the manuscript. All authors read and approved the final manuscript.

## Pre-publication history

The pre-publication history for this paper can be accessed here:

http://www.biomedcentral.com/1472-6831/14/37/prepub

## References

[B1] Sugai M Kawamoto T Pérès SY Ueno Y Komatsuzawa H Fujiwara T Kurihara H Suginaka H Oswald E The cell cycle-specific growth-inhibitory factor produced by *Actinobacillus actinomycetemcomitans* is a cytolethal distending toxin Infect Immun 1998 66 5008 5019 9746611 10.1128/iai.66.10.5008-5019.1998 PMC108622

[B2] Asakura M Samosornsuk W Taguchi M Kobayashi K Misawa N Kusumoto M Nishimura K Matsuhisa A Yamasaki S Comparative analysis of cytolethal distending toxin (cdt) genes among *Campylobacter jejun*i, *C. coli* and *C. fetus* strains Microb Pathog 2007 42 174 183 10.1016/j.micpath.2007.01.005 17353111

[B3] Fox JG Rogers AB Whary MT Ge Z Taylor NS Xu S Horwitz BH Erdman SE Gastroenteritis in NF-kappaB-deficient mice is produced with wild-type *Camplyobacter jejuni* but not with C. jejuni lacking cytolethal distending toxin despite persistent colonization with both strains Infect Immun 2004 72 1116 1125 10.1128/IAI.72.2.1116-1125.2004 14742559 PMC321575

[B4] Ahmed HJ Svensson LA Cope LD Latimer JL Hansen EJ Ahlman K Bayat-Turk J Klamer D Lagergård T Prevalence of cdtABC genes encoding cytolethal distending toxin among *Haemophilus ducreyi* and *Actinobacillus actinomycetemcomitans strains* J Med Microbiol 2001 50 860 864 11599734 10.1099/0022-1317-50-10-860

[B5] Haghjoo E Galán JE *Salmonella typhi* encodes a functional cytolethal distending toxin that is delivered into host cells by a bacterial-internalization pathway Proc Natl Acad Sci U S A 2004 101 4614 4619 10.1073/pnas.0400932101 15070766 PMC384795

[B6] Lara-Tejero M Galán JE A bacterial toxin that controls cell cycle progression as a deoxyribonuclease I-like protein Science 2000 290 354 357 10.1126/science.290.5490.354 11030657

[B7] Elwell CA Dreyfus LA DNase I homologous residues in CdtB are critical for cytolethal distending toxin-mediated cell cycle arrest Mol Microbiol 2000 37 952 963 Ge Z, Schauer DB, Fox JG.: In vivo virulence properties of bacterial cytolethal-distending toxin. *Cell Microbiol* 2008, **10:** 1599-1607. 10.1046/j.1365-2958.2000.02070.x 10972814

[B8] Fabris AS DiRienzo JM Wïkstrom M Mayer MP Detection of cytolethal distending toxin activity and cdt genes in *Actinobacillus actinomycetemcomitans* isolates from geographically diverse populations J Oral Microbiol Immunol 2002 17 231 238 10.1034/j.1399-302X.2002.170405.x PMC2548306 12121473

[B9] Yamano R Ohara M Nishikubo S Fujiwara T Kawamoto T Ueno Y Komatsuzawa H Okuda K Kurihara H Suginaka H Oswald E Tanne K Sugai M Prevalence of cytolethal distending toxin production in periodontopathogenic bacteria J Clin Microbiol 2003 41 1391 1398 10.1128/JCM.41.4.1391-1398.2003 12682119 PMC153874

[B10] Kawamoto D Ando ES Longo PL Nunes AC Wikström M Mayer MP Genetic diversity and toxic activity of *Aggregatibacter actinomycetemcomitans* isolates Oral Microbiol Immunol 2009 24 493 501 10.1111/j.1399-302X.2009.00547.x 19832802

[B11] Armitage GC Development of a classification system for periodontal diseases and conditions Ann Periodontol 1999 4 1 6 10.1902/annals.1999.4.1.1 10863370

[B12] American Academy of Periodontology Parameter on progressive periodontitis J Periodontol 2000 71 867 869 10875695 10.1902/jop.2000.71.5-S.867

[B13] Meng S Zhao L Yang H Wu Y Ouyang Y Prevalence of *Actinobacillus actinomycetemcomitans* in Chinese chronic periodontitis patients and periodontally healthy adults Quintessence Int 2009 40 53 60 19159024

[B14] Cortelli SC Costa FO Kawai T Aquino DR Franco GC Ohara K Roman-Torres CV Cortelli JR Diminished treatment response of periodontally diseased patients infected with the JP2 clone of *Aggregatibacter (Actinobacillus) actinomycetemcomitans* J Clin Microbiol 2009 47 2018 2025 10.1128/JCM.00338-09 19458180 PMC2708506

[B15] Jervøe-Storm PM Alahdab H Koltzscher M Fimmers R Jepsen S Comparison of curet and paper point sampling of subgingival bacteria as analyzed by real-time polymerase chain reaction J Periodontol 2007 78 909 917 10.1902/jop.2007.060218 17470026

[B16] Krigar DM Kaltschmitt J Krieger JK Eickholz P Two subgingival plaque sampling strategies used with RNA-probes J Periodontol 2007 78 72 78 10.1902/jop.2007.060236 17199542

[B17] Nonnenmacher C Dalpke A Mutters R Heeg K Quantitative detection of periodontopathogens by real-time PCR J Microbiol Methods 2004 59 117 125 10.1016/j.mimet.2004.06.006 15325758

[B18] Cortelli JR Roman-Torres CV Aquino DR Franco GC Costa FO Cortelli SC Occurrence of *Aggregatibacter actinomycetemcomitans* in Brazilians with chronic periodontitis Braz Oral Res 2010 24 217 223 10.1590/S1806-83242010000200015 20658042

[B19] Turenne CY Tschetter L Wolfe J Kabani A Necessity of quality-controlled 16S rRNA gene sequence databases: identifying nontuberculous Mycobacterium species J Clin Microbiol 2001 39 3637 3648 10.1128/JCM.39.10.3638-3648.2001 11574585 PMC88401

[B20] Yoshida A Suzuki N Nakano Y Oho T Kawada M Koga T Development of a 5′ fluorogenic nuclease-based real-time PCR assay for quantitative detection of *Actinobacillus actinomycetemcomitans* and *Porphyromonas gingivalis* J Clin Microbiol 2003 41 863 866 10.1128/JCM.41.2.863-866.2003 12574302 PMC149717

[B21] Morillo JM Lau L Sanz M Herrera D Martín C Silva A Quantitative real-time PCR based on single copy gene sequence for detection of *Actinobacillus actinomycetemcomitans* and *Porphyromonas gingivalis* J Periodontal Res 2003 38 518 524 10.1034/j.1600-0765.2003.00684.x 12941077

[B22] Doungudomdacha S Rawlinson A Douglas CW Enumeration of Porphyromonas gingivalis, Prevotella intermedia and Actinobacillus actinomycetemcomitans in subgingival plaque samples by a quantitative-competitive PCR method J Med Microbiol 2000 49 861 874 11023183 10.1099/0022-1317-49-10-861

[B23] Tan KS Song KP Ong G Cytolethal distending toxin of Actinobacillus actinomycetemcomitans.:Occurrence and association with periodontal disease J Periodontal Res 2002 37 268 272 10.1034/j.1600-0765.2002.01618.x 12200970

[B24] Casarin RC Ribeiro Edel P Mariano FS Nociti FH Jr Casati MZ Gonçalves RB Levels of aggregatibacter actinomycetemcomitans, porphyromonas gingivalis, inflammatory cytokines and species-specific immunoglobulin G in generalized aggressive and chronic periodontitis J Periodontal Res 2010 45 635 642 10.1111/j.1600-0765.2010.01278.x 20546109

[B25] Kaplan JB Schreiner HC Furgang D Fine DH Population structure and genetic diversity of Actinobacillus actinomycetemcomitans strains isolated from localized juvenile periodontitis patients J Clin Microbiol 2002 40 1181 1187 10.1128/JCM.40.4.1181-1187.2002 11923328 PMC140340

[B26] Schacher B Baron F Rossberg M Wohlfeil M Arndt R Eickholz P Aggregatibacter actinomycetemcomitans as indicator for aggressive periodontitis by two analyzing strategies J Clin Periodontol 2007 34 566 573 10.1111/j.1600-051X.2007.01080.x 17433043

